# New Insight into Laboratory Tests and Imaging Modalities for Fast and Accurate Diagnosis of COVID-19: Alternative Suggestions for Routine RT-PCR and CT—A Literature Review

**DOI:** 10.1155/2020/4648307

**Published:** 2020-11-28

**Authors:** Amir Khorasani, Amir Chegini, Arezoo Mirzaei

**Affiliations:** ^1^Department of Medical Physics, Faculty of Medicine, Isfahan University of Medical Science, Isfahan, Iran; ^2^Faculty of Medicine, Semnan University of Medical Science, Semnan, Iran; ^3^Department of Bacteriology and Virology, Faculty of Medicine, Isfahan University of Medical Science, Isfahan, Iran

## Abstract

The globally inimitable and unremitting outbreak of COVID-19 infection confirmed the emergency need for critical detection of human coronavirus infections. Laboratory diagnostic tests and imaging modalities are two test groups used for the detection of COVID-19. Nowadays, real-time polymerase chain reaction (RT-PCR) and computed tomography (CT) have been frequently utilized in the clinic. Some limitations that confront with these tests are false-negative results, tests redone for follow-up procedure, high cost, and unable to do for all patients. To overcome these limitations, modified and alternative tests must be considered. Among these tests, RdRp/Hel RT-PCR assay had the lowest diagnostic limitation and highest sensitivity and specificity for the detection of SARS-CoV-2 RNA in both respiratory tract and nonrespiratory tract clinical specimens. On the other hand, lung ultrasound (LUS) and magnetic resonance imaging (MRI) are CT-alternative imaging modalities for the management, screening, and follow-up of COVID-19 patients.

## 1. Introduction

**Figure 1:**
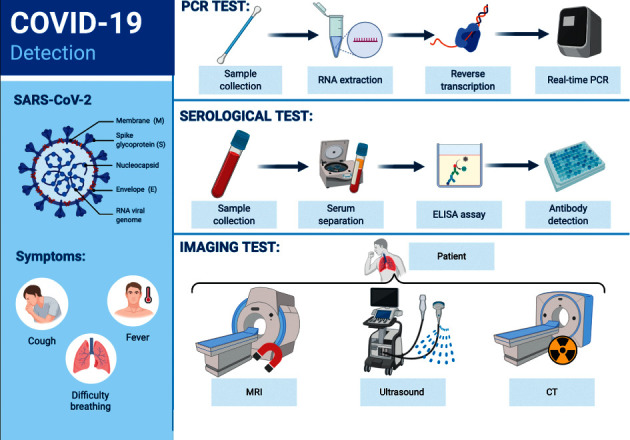
Summarized COVID-19 detection tests.

The disease that makes with new emerging coronaviruses belongs to acute respiratory disease (ARD), which causes many acute morbidities in developed countries, and to date, most patients with COVID-19 have developed mild symptoms such as dry cough, sore throat, and fever. The large part of the cases automatically improved. Nevertheless, some parts developed various lethal complications counting organ failure, septic shock, pulmonary edema, severe pneumonia, and acute respiratory distress syndrome (ARDS) [1]. Respiratory viruses could be transmitted by such routes, including large droplets, aerosols, or fomites (objects or surfaces that may have the virus) that cause the direct inoculation of either the nasal or conjunctival epithelium [2]. The notable points for controlling the outbreak in different settings are these modes of transmission. Our understanding of the epidemiology of respiratory virus infections is changing with the discovery of new viruses introduced into the human population and new emerging identified viruses (SARS-CoV-2) that nowadays are circulating in humans for some time and make epidemic such as the COVID-19 outbreak. The tests used for the detection of COVID-19 are divided into two groups, including (1) laboratory diagnostic test and (2) imaging modality (Figure 1). This outbreak had a massive impact on clinicians and clinical microbiology labs in the past months, in which emergency need for appropriate measures feels to accompany with the utilization of more sensitive imaging techniques and molecular tests for their detection. Rapid etiological diagnosis of respiratory virus infection may influence antiviral and antibiotic therapy, patient groups, and prediction of the clinical period. The first laboratory methods, which are conventional diagnostic tests for respiratory virus infections, include cell culture for virus isolation and serology, but for this viral respiratory pandemic, viral cultures are not recommended. Serological tests were other tests that are carried out, which contain the hemagglutination inhibition (HAI) test, complement fixation, and EIA. Shell vial culture (SVC) was established at the first of the 1990s, and, along with the evolution of specific monoclonal antibodies, viral antigens could be detected in cell culture at a faster time (1-2 days) in lieu the routine 8–10 days for tube culture. At the same time, in the 1990s, another rapid test, DFA (direct fluorescent antibody) staining of nasopharyngeal (NP) swabs or NP aspirates, with the time consumption of about 3 hours, became the central point for many laboratories. Such point-of-care (POC) tests, which were rapid for diagnosing of viral antigens, with the name EIA tests, were introduced in the 1980s and 1990s, but these point-of-care tests (POCT) had a low sensitivity. Most point-of-care tests for the detection of respiratory viruses have limitations in diagnostic performance and clinical usability [2, 3].

Nevertheless, these tests have been widely used in some settings, but in some situations, low sensitivity of tests affects the diagnosis and identification of patients, and they should be transferred to settings with a high pretest probability. The pretest probability is a clinician's best estimate of the probability of disease in a group of individuals with similar symptoms [4]. Now, computed tomography (CT) is widely used as the imaging modality for the detection of COVID-19 pneumonia. The fast way in order to imaging that is currently used in hospitals is CT scan. CT for some people such as pregnant women and older adults do not become useable for monitoring and screening goals because the use of ionizing radiation is not recommended. So, we should find a CT-alternative imaging modality for patients with COVID-19, which utilized nonionizing radiation in order to screening, monitoring, and follow-up purposes. Magnetic resonance imaging (MRI) and ultrasound imaging are the most useful imaging modalities which employed nonionization radiation for imaging acquisition. This exegesis represents current subject matters for the diagnosis of COVID-19 and also alternative ways for CT scan that must be noticed and understood both by clinicians, clinical microbiology laboratories, and public health specialists.

## 2. Main Text

### 2.1. Sample Collection

5 to 6 days after the onset of a symptom of COVID-19, the high load of the virus could be detected in the lower and upper respiratory tract [5–7]. Suggested swabs for screening or early diagnosis are nasopharyngeal and oropharyngeal swabs, which are better to be flocked, nontoxic polyester or synthetic nylon [8], and the former is the preferred swab because it could be better tolerated by patients and also safer for the operator [6]. For patients with pneumonia, in addition to nasopharyngeal swabs, lower respiratory tract secretions, including sputum and bronchoalveolar lavage fluid, are tested [9]. However, there are some points and rules that must be noticed until the swab reaches exactly correct place in the nasal cavity. The RNA of the SARS-CoV-2 virus is isolated in just 32% of OP swabs that is significantly lower than nasal swabs, with 63% [9]. So, for best form doing sampling, swabs should be deeply inserted in the nasal cavity until it makes tears; this makes sure that hits the target. Also, swabs must hold in the target place for 10 seconds with three times of twirling [8]. If there are any qualified equipment, it is better to use an alternative way of collecting the upper respiratory tract samples, which are for suspected patients with pneumonia saliva self-collected samples [10–12]. In some cases, with NP, OP, and saliva samples, we can lose primary infection, which may need repetitive tests or sampling from the lower respiratory tract [4].

Repetitive tests might be necessary if a patient had a clinical image of viral pneumonia or a history of exposure or radiologic finding that is consistent with COVID-19 pneumonia. The ideal samples for lower respiratory tracts are sputum or bronchoalveolar lavage because they have a higher load of virus for COVID-19 detection [9, 13]. The past studies demonstrate that bronchoalveolar lavage had the most RNA rate for SARS-CoV-2 [14, 15]. Overall, there are two types of sampling, direct respiratory sampling and indirect, in which the preferred method for SARS-CoV-2 identification is the first type. In some progressive conditions of COVID-19, rectal swabs could be used for RT-PCR [16–19].

### 2.2. Serologic Diagnosis

Serology measurement is a host response to infection and counts an indirect measurement. Those tests rapidly improved and became useful for COVID-19 confirmation [20, 21]. Multiple serological immunoassay methods developed by companies for detection of SARS-CoV2 proteins and antibodies in serum or plasma include as follows:

1. Rapid lateral flow immune assay (LFIA)
2. Automated chemiluminescence immune assay (CLIA)
3. Manual ELISA

The first method of the list for both IgM and IgG antibodies undoubtedly plays a vital role in COVID-19. IgM response is nonspecific and needs multiple weeks to develop an IgG response, which could reside for a long time after the infection and also makes a protective role. The tests which detect the polyclonal antibodies in patients against SARS-CoV-2 are faster to expand other than detection tests that identified the virus [22]. Also, the serological tests, combined with immunochromatography, colloidal gold, and other technologies, have been developed rapidly [20].

### 2.3. Nucleic Acid-Based Diagnosis

The most common test used for diagnosis of COVID-19 is viral RNA identification by nucleic acid amplification, that is, by PCR [13, 23, 24]. Random amplification deep sequencing method plays a vital role in the initial detection of SARS-CoV-2. Deep sequencing molecular methods such as next generation sequencing and metagenomic NGS could define future mutations, but this method is impractical for the detection of patients with COVID-19 [25–28]. The test has been broadly used for confirmation of this infection based on RT-PCR. Coronaviruses have some molecular targets that could be used for PCR assay, which include RNA-dependent RNA polymerase (RdRp), hemagglutinin esterase (HE), ORF1a, and ORF1b [29].

For PCR targeting in the United States, CDC suggests two nucleocapsid proteins (N1 and N2) [30], while WHO announced the first line of screening with E gene assay, which is followed by confirmatory identification with the RdRp gene [31]. Many tests with variation in targets have been done, but among them, RdRp/Hel assay had in vitro lowest diagnostic limitation and highest sensitivity and specificity [29]. Also, a rapid detection test has been invented named nucleic acid test paper that could be used for SARS-CoV2 with naked eyes within three minutes [32]. Nevertheless, until now, there is no sign of observation that these sequences have advantages for clinical detection. So still, an ideal design contains at least one conserve and a specific region with a reduction influence on genetic drift on them. However, the primary concern about the nucleic acid-based methods is false-negatives [33].

### 2.4. CRISPR/Cas System

CRISPR is a biotechnological technique for gene editing which nowadays has been used for the detection of nucleic acids, so a precise and powerful tool in molecular detection is emerging. With Cas 12 and 13-based SHERLOCK (specific high-sensitivity enzymatic reporter unlocking) platform and combination of the Cas, nucleic acid detection was performed, the latter has been widely used to detect the Zika virus (ZIKV) and dengue virus (DENV) in patient samples with minimum concentration load of the virus as low as one copy per microliter [34] and former Cas that is an RNA-guided DNase, ssDNA parallel cleavage induced after a target recognition which results in the deletion of ssDNA reporters. This deletion emits a fluorescence signal that could be identified on a paper strip in a portable path. With the use of CRISPR SHERLOCK technology, from synthetic SARS-CoV-2 RNA virus segments, a target sequence of COVID-19 could be identified in a range of 10–100 copies per microliter. Furthermore, this test would be done with a dipstick in less than an hour [35].

### 2.5. Imaging Modality

Chest radiograph (CXR) or computed tomography (CT) is an essential tool for COVID-19 diagnosis in clinical practice. The majority of COVID-19 cases have similar features on CT images, including bilateral distribution of patchy shadows and ground-glass opacity [36]. CXR and chest CT expose the patient to ionizing radiation. In order to monitoring and follow-up, patients may receive multiple CXR and chest CT. On the other hand, ionization radiation is harmful and Increases the risk of later cancers [37–39] and other complications especially in sensitive people such as fetus, children, and older person so that we would be able to use different imaging modalities which use nonionizing radiation such as ultrasound imaging and magnetic resonance imaging (MRI).

### 2.6. Ultrasound Imaging

One of the chest CT and CXR-alternative imaging modality is ultrasound sonography. Previous works have focused on representing lung ultrasound (LUS) as a diagnostic technique for diagnosing pulmonary and pleural diseases [40–43]. For the detection of pneumonia patients with consolidation or pleural effusion, the sensitivity of LUS, CXR, and chest CT are the same [44]. Reissig et al. [45] have demonstrated the sensitivity and specificity of lung ultrasound (LUS) for diagnosis of community-acquired pneumonia (CAP) which is 93.4% and 97.7%, respectively.

More recent evidence highlights the benefits of LUS for COVID-19. Chest CT and LUS images showed a strong correlation in COVID-19 pneumonia [46]. Obstetricians can learn how to perform lung ultrasound for COVID-19 detection with minimal training and measurement by obstetricians showing good agreement with radiology specialists [47]. Diffuse B-pattern and subpleural consolidation with different patterns; thickening of pleural line, focal, multifocal, and confluent B-lines; A-lines appearance in the recovery phase; and mixed pattern with A- and B-lines were detected in LUS images of patients with COVID-19 pneumonia [46, 48, 49]. In Italy, Soldati et al. [50] used a lung ultrasound of 30 confirmed COVID-19 cases in order to introduce acquisition protocol and scoring procedures of COVID-19 pneumonia. Their research has highlighted the critical role of lung ultrasound sonography on the early diagnosis and monitoring and management of the patient with SARS-CoV-2.

Lung ultrasound in pregnant women is easy to perform and effective in assessing lung involvement [51]. Anecdotal evidence suggests that lung involvement in pregnant women is readily seen with lung ultrasound and can influence the clinical management of pregnant women with COVID-19 [52–54].

### 2.7. Magnetic Resonance Imaging

Magnetic resonance imaging (MRI) is a diagnostic imaging modality with high soft-tissue contrast, ability to characterize tissue properties, functional imaging, and used nonionizing radiation. However, lung MRI is not clinically well-used because of some limitations [55]. Lung MRI is limited by low proton density, long acquisition time, and rapid signal decay of region of interest, susceptibility artifacts at the air-tissue interface, and motion artifacts of cardiac and respiratory motions [55]. Some researches were carried out to overcome these limitations [56–59]. A recent study on this subject [59] found that, for imaging pulmonary lesions induced by tuberculosis (TB), we can use the MultiVane motion correction technique. With this optimized MRI protocol, they offer an alternative imaging technique to the clinical standard CT for diagnosis and characterization of pulmonary lesions. More recent evidence [57] shows the critical role of lung MRI on the determination of pulmonary lesions. Rana et al. [57] reported the specificity and sensitivity of lung MRI in HIV-positive children. They concluded that lung MRI for detecting nodules >4 mm is a high sensitivity and specificity modality. More recent evidence [56] shows that contrast-enhanced free-breathing stack-of-stars GRE (starVIBE) MRI pulse sequence can produce high spatial and temporal resolution and high-quality dynamic images in patients with idiopathic pulmonary fibrosis (IPF) with excellent correlation to CT. Many studies have been published on the comparison of thoracic MRI to CT [60–62]. They point out that all patients with pleural effusion, consolidation, and pulmonary cyst were seen on both CT and MRI [60–63]. In comparison with CT, halo sign and ground-glass opacities (GGO) were detected in 88% and 64% in MRI images, respectively [64].

## 3. Conclusion

The globally inimitable and unremitting outbreak of COVID-19 infection confirmed the emergency need for critical detection of human coronavirus infections. As mentioned above, the tests used for the detection of COVID-19 are divided into two groups, including (1) laboratory diagnostic test and (2) imaging modality. The critical point that must be noticed before the laboratory tests is a correct and precise sampling. Suggested swabs for screening or early diagnosis are nasopharyngeal and oropharyngeal swab, which is better to be flocked, nontoxic polyester or synthetic nylon. In lab methods, there is a gold standard for detection which is RT-PCR, but the use of it has some challenges in which the most vital of them is false-negative results. To overcome this issue, the scientists that have done in vitro experiments get to this result that RdRp/Hel assay had lowest diagnostic limitation and highest sensitivity and specificity for detection of SARS-CoV-2 RNA in both respiratory tract and nonrespiratory tract clinical specimens. Also, among the POC tests which have been done for primary screening, the combination of Cas 12 and Cas 13 SHERLOCK technology could recognize the target sequence of COVID-19 in a range of 10–100 copies per microliter with the dipstick in less than an hour. Moreover, about the serological tests, studies demonstrate that the polyclonal antibodies in patients against SARS-CoV-2 quickly develop other than the detection of the virus alone, as well as the combination of serological tests with immunochromatography, colloidal gold, and other technologies, has been developed rapidly.

On the other hand, in imaging modality, we can use chest CT, CXR, LUS, lung MRI, and nuclear medicine as a diagnostic tool for COVID-19 detection. We have demonstrated that LUS is a good CXR and chest CT-alternative modality for the management of COVID-19 patients due to its absence of ionizing radiation, portability, low cost, safety, repeatability, and wide availability. So, the use of LUS was strongly recommended for early detection of COVID-19 pneumonia for all the patients with novel COVID-19 symptoms. Elseways, MRI has the potential to monitoring and follow-up on COVID-19 patients because of the advantage of using nonionizing radiation, high soft-tissue contrast, functional imaging, and high sensitivity and specificity for lung lesions. The present findings have important implications for solving the monitoring and follow-up issues in COVID-19 patients. In this paper, we have suggested the imaging modality with nonionizing radiation, which we can use as management, monitoring, and follow-up tools for COVID-19 patients. Also, in our view, this imaging modality could be applied to radiation-sensitive patients such as pregnant, fetus, children, and old patients with COVID-19 symptoms. It probably seems that the combination of two or more methods could help to our goal that is the precise and rapid identification test with the lowest error. The precise and rapid etiological diagnosis of respiratory virus infection may influence antiviral and antibiotic therapy, patient groups, and prediction of the clinical period.

## Data Availability

The data supporting this study are from previously reported studies and datasets, which have been cited.
